# The Roles of Exosomes in Anti‐Cancer Drugs

**DOI:** 10.1002/cam4.70897

**Published:** 2025-04-29

**Authors:** Panpan Feng, Xiaodong Zhang, Jian Gao, Lei Jiang, Yan Li

**Affiliations:** ^1^ Department of Radiotherapy The First Affiliated Hospital of Jinzhou Medical University Jinzhou China; ^2^ Department of General Surgery Beijing Friendship Hospital, Capital Medical University Beijing China; ^3^ Science Experiment Center of China Medical University Shenyang China; ^4^ Department of General Surgery The First Affiliated Hospital of Jinzhou Medical University Jinzhou China; ^5^ Liaoning Provincial Key Laboratory of Clinical Oncology Metabonomics The First Affiliated Hospital of Jinzhou Medical University Jinzhou China

**Keywords:** cancer, chemotherapy, drug carrier, exosome

## Abstract

**Background:**

Cancer is an escalating global health issue, with rising incidence rates annually. Chemotherapy, a primary cancer treatment, often exhibits low tumor‐targeting efficiency and severe side effects, limiting its effectiveness. Recent research indicates that exosomes, due to their immunogenicity and molecular delivery capabilities, hold significant potential as drug carriers for tumor treatment.

**Methods:**

This review summarizes the current status, powerful therapeutic potential, and challenges of using exosomes for the treatment of tumors.

**Results:**

Exosomes are crucial in tumor diagnosis, onset, and progression. To improve the efficacy of exosome‐based treatments, researchers are exploring various biological, physical, and chemical approaches to engineer exosomes as a new nanomedicine translational therapy platform with broad and alterable therapeutic capabilities. Numerous clinical trials are currently underway investigating the safety and tolerability of exosomes carrying drugs to specific sites for the treatment of tumors.

**Conclusions:**

Exosomes can be engineered as carriers to deliver therapeutic molecules to specific cells and tissues, offering a novel approach for disease treatment.

## 1 Background

Cancer is a multifaceted and diverse disease influenced by numerous factors, characterized by resistance to cell death, altered metabolism, sustained growth signaling, evasion of growth suppression, immune evasion, limitless replication, promotion of tumor inflammation, activation of invasion and metastasis, induction of angiogenesis, genomic instability and mutations, epigenetic reprogramming, diverse microbial communities, unlocked phenotypic plasticity, and senescent cells [1]. Various treatment methods for malignant tumors, such as surgery, chemotherapy, radiation therapy, targeted therapy, and immunotherapy, have been developed based on their unique characteristics. Chemotherapy is a type of chemical drug treatment that involves the use of chemical agents to prevent, impede, or eliminate cancer cells from growing, infiltrating, and metastasizing [2]. It can serve as a standalone systemic therapy for malignancies or be combined with treatments like radiation and immunotherapy. Its benefits include the ability to eliminate cancer cells systemically and inhibit early‐stage metastasis. Nevertheless, its drawbacks encompass significant systemic side effects, partial intolerance to complete treatment by certain patients, and unclear targeting specificity. Despite advances in cancer treatment, malignant tumors remain among the leading causes of human death. Cancer cells often exhibit drug resistance, leading to poor prognosis or treatment outcomes. This resistance arises from mechanisms such as increased drug efflux, reduced drug uptake, drug inactivation, target mutations, altered signaling pathways, apoptosis defects, and phenotypic changes [3]. Another factor contributing to the limited effectiveness of chemotherapy is the inability of drugs to reach specific regions within tumors [4]. Thus, overcoming drug resistance in tumor cells and delivering therapeutic agents to specific sites using specific carriers are crucial steps for improving cancer treatment efficacy.

Exosomes are membrane‐bound structures, 40–100 nm in diameter, secreted by diverse cell types under both physiological and pathological conditions [5]. Exosomes are released into the extracellular matrix following their fusion with the cell membrane. Exosomes carry diverse substances, including DNA, RNA, and proteins, with their functions determined by their specific cargo [6]. Exosomes can transport various forms of DNA, including single‐stranded, double‐stranded, and mitochondrial DNA, along with non‐coding RNAs like microRNA (miRNA), messenger RNA (mRNA), long non‐coding RNA (lncRNA), and circular RNA (circRNA) [7, 8]. Exosomes have shown great potential in cell communication, angiogenesis, antigen presentation, cancer invasion and metastasis, tumor microenvironment remodeling, drug delivery, and other aspects, due to their ability to deliver these substances [9]. Structurally, exosomes resemble lipid nanoparticles, which can protect and transport internal components, making them ideal drug carriers [10, 11]. Significant advancements have been achieved in utilizing exosomes as drug carriers for targeted delivery and treatment of tumor‐specific sites, as research on exosomes and cancer therapy progresses.

This article focuses on the current research status of exosomes, including their sources, characteristics, functions, clinical applications, and use as drug carriers for cancer treatment. The challenges and future development trends in this area are also discussed to facilitate further developments in related fields.

## 2 Main Text

### 2.1 The Properties of Exosomes

#### 2.1.1 Formation of Exosomes

Exosomes were first discovered in sheep reticulocytes in 1983 [12]. They facilitate intercellular signaling by being released into the extracellular space following the fusion of multivesicular bodies (MVBs) with the cell membrane [13]. Extracellular vesicles are categorized by size into exosomes (30–100 nm), microvesicles (100–1000 nm), and apoptotic bodies (10–5000 nm) [14].

**FIGURE 1:**
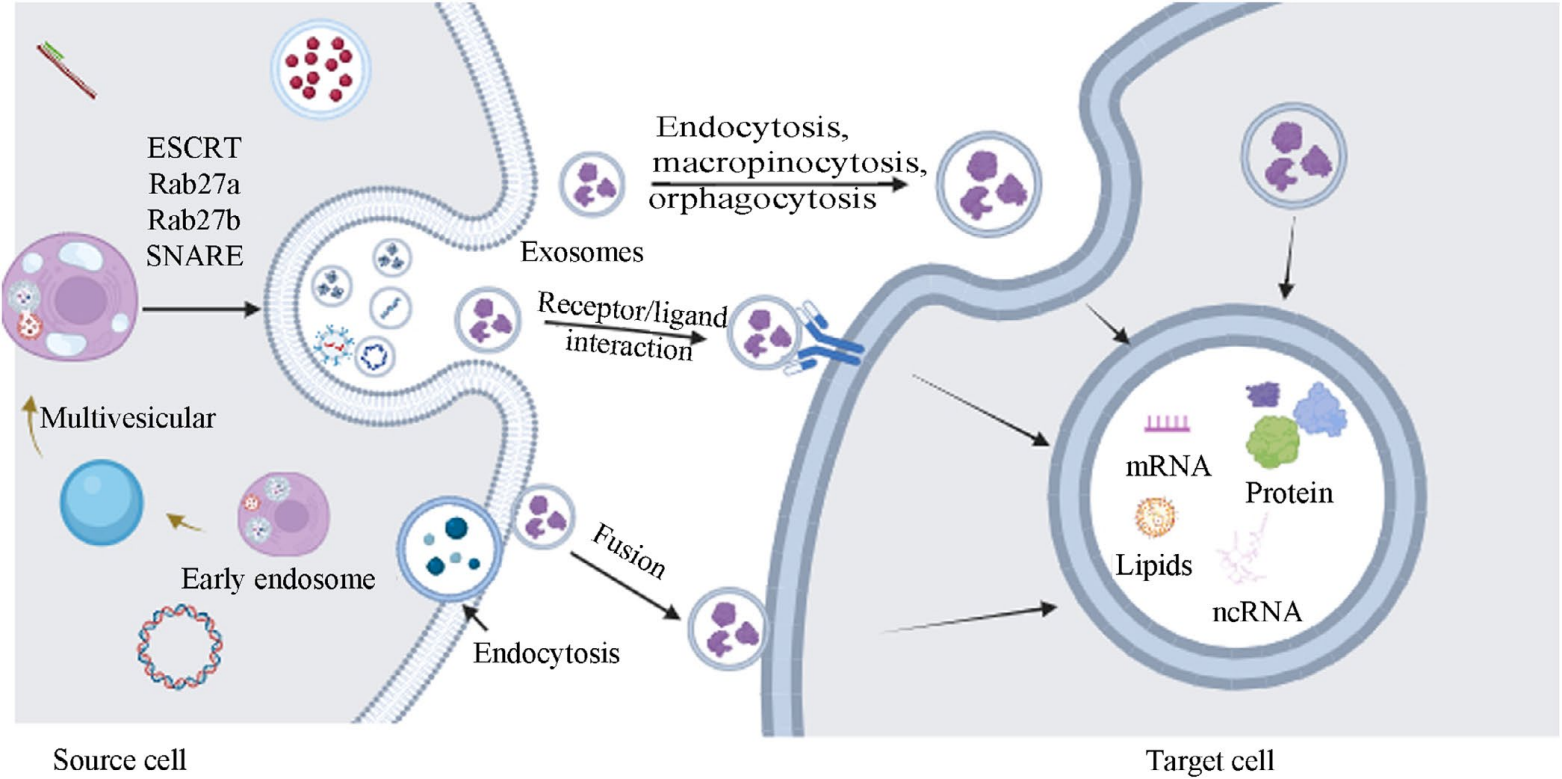
Overview of exosome biogenesis and target cells uptake of exosomes.

Exosomes formation is a multifaceted process comprising three stages: cell membrane invagination to create intraluminal vesicles, inward budding of these vesicle membranes to develop multivesicular bodies, and the generation of intraluminal vesicles within these bodies [15] (Figure 1). Prior research indicates that the endosomal sorting complex required for transport (ESCRT) plays a role in the formation and release of exosomes [16]. ESCRT is composed of ESCRT0, ESCRT1, ESCRT2, ESCRT3, and vacuolar protein sorting‐associated protein 4 (VPS4) [17]. The internalization of ubiquitinated proteins is initiated by ESCRT0, followed by ESCRT1 and ESCRT2‐mediated inward vesicle budding to form MVBs. Certain MVBs are degraded by lysosomes, whereas others merge with the cell membrane and are released into the extracellular space through the action of ESCRT3 and VPS4 ATPase.

In addition to this pathway, there are other ways to form exosomes. For example, Rab proteins regulate the occurrence of exosomes through the regulation of the nuclear envelope and plasma membrane [18]. Rab27a and Rab27b participate in the generation and localization of MVBs [19]. Rab27a organizes plasma membrane microdomains by modulating PIP2 to facilitate the budding of vesicles.

Overall, the formation and function of extracellular vesicles are complex processes that involve various regulatory factors. Further research into these processes could lead to the development of new diagnostic and therapeutic strategies for various diseases.

#### 2.1.2 Release of Exosomes

Exosome release into the extracellular space involves crucial steps mainly during the separation of multivesicular bodies (MVBs) from the cell membrane [20]. Previous studies have confirmed that GTPases are the most critical factors mediating exosome release. Research has identified nine GTPases involved in exosome release: Rab2B, Rab5, Rab7, Rab9A, Rab11, Rab27A, Rab27B, Rab35, and RAL [21, 22, 23]. Rab is essential for directing MVBs to specific subcellular sites and facilitating their fusion with the cell membrane. Studies indicate that MVBs can associate with actin and microtubule cytoskeletons, either directly or indirectly, to facilitate targeted intracellular transport [24]. Rab11 and its related proteins facilitate MVB transport via actin and motor proteins [25]. Moreover, Rab27A, along with myosin, has been shown to transport melanosomes along actin filaments [26]. Overall, the process from MVBs to exosomes not only relies on the action of motor proteins but also requires the separation of MVBs from the cell membrane.

The SNARE complex is crucial for MVB fusion with the cell membrane, facilitating their release. The SNARE complex consists of two components, *v*‐SNARE and *t*‐SNARE. V‐SNARE resides on the vesicle, whereas *t*‐SNARE is found on the presynaptic membrane [27]. During the formation of the complex, the released energy brings the MVB closer to the presynaptic membrane, promoting MVB membrane fusion. Research indicates that VAMP7, a *v*‐SNARE protein, is involved in MVB membrane fusion and exosomes release in K562 cells [28]. In mammals, SYX‐5 (one of the *t*‐SNARE proteins) also participates in MVBs fusion to promote exosomes release [29]. In tumor cells that primarily depend on aerobic glycolysis, the glycolytic enzyme PKM2 enhances exosomes release by stabilizing the SNARE complex through phosphorylation of the *t*‐SNARE protein SNAP‐23 [30]. Research suggests that exosomes release mediation is a multifaceted process with various steps and factors involved (Figure 1). Despite variations in regulatory mechanisms across cell types, GTPases and the SNARE complex are commonly involved in the primary processes. Additional studies are required to verify if intervening in the synthesis of these two components can effectively regulate exosomes release.

#### 2.1.3 Uptake of Exosomes

Once released into the extracellular environment, exosomes can be re‐absorbed and utilized by their own cells but can be taken up by neighboring cells. Exosomes primarily enter target cells through membrane fusion, endocytosis, or by binding to specific surface receptors, thereby facilitating intercellular communication [31]: (Figure 1) membrane fusion, endocytosis, and binding with specific surface receptors of target cells to facilitate intercellular communication (Figure 1). Currently, the main factors that determine exosome uptake are not fully understood. Edgar et al. suggested that tetherin protein could mediate exosomes attachment to the cell surface and affect exosomes signaling transduction [32]. Christianson et al. found that heparan sulfate proteoglycans (HSPGs) on the surface of recipient cells not only serve as attachment sites for exosomes but also act as receptors for exosomes uptake [33]. During exosome uptake, proteins that promote or inhibit exosome uptake coexist. For example, CD47 molecules on the exosome surface usually protect exosomes from being phagocytosed by recipient cells, thereby enhancing their presence in the microenvironment [34]. In summary, although there has been considerable research on exosome uptake, the underlying mechanisms and factors influencing this process remain incompletely understood. Given the vital role of exosomes in intercellular communication, additional research is necessary to clarify the intricate interactions between exosomes and recipient cells.

#### 2.1.4 Extraction Methods of Exosomes

Exosomes are naturally found in body fluids such as blood, urine, saliva, cerebrospinal fluid, and breast milk, and can be secreted by nearly all cell types. Improving the efficiency of exosome extraction is a primary concern for their clinical application. Although the mechanisms underlying exosome extraction and cargo loading are still under investigation, the currently mature techniques for exosome extraction mainly include the following.

#### 2.1.5 Ultracentrifugation

**FIGURE 2:**
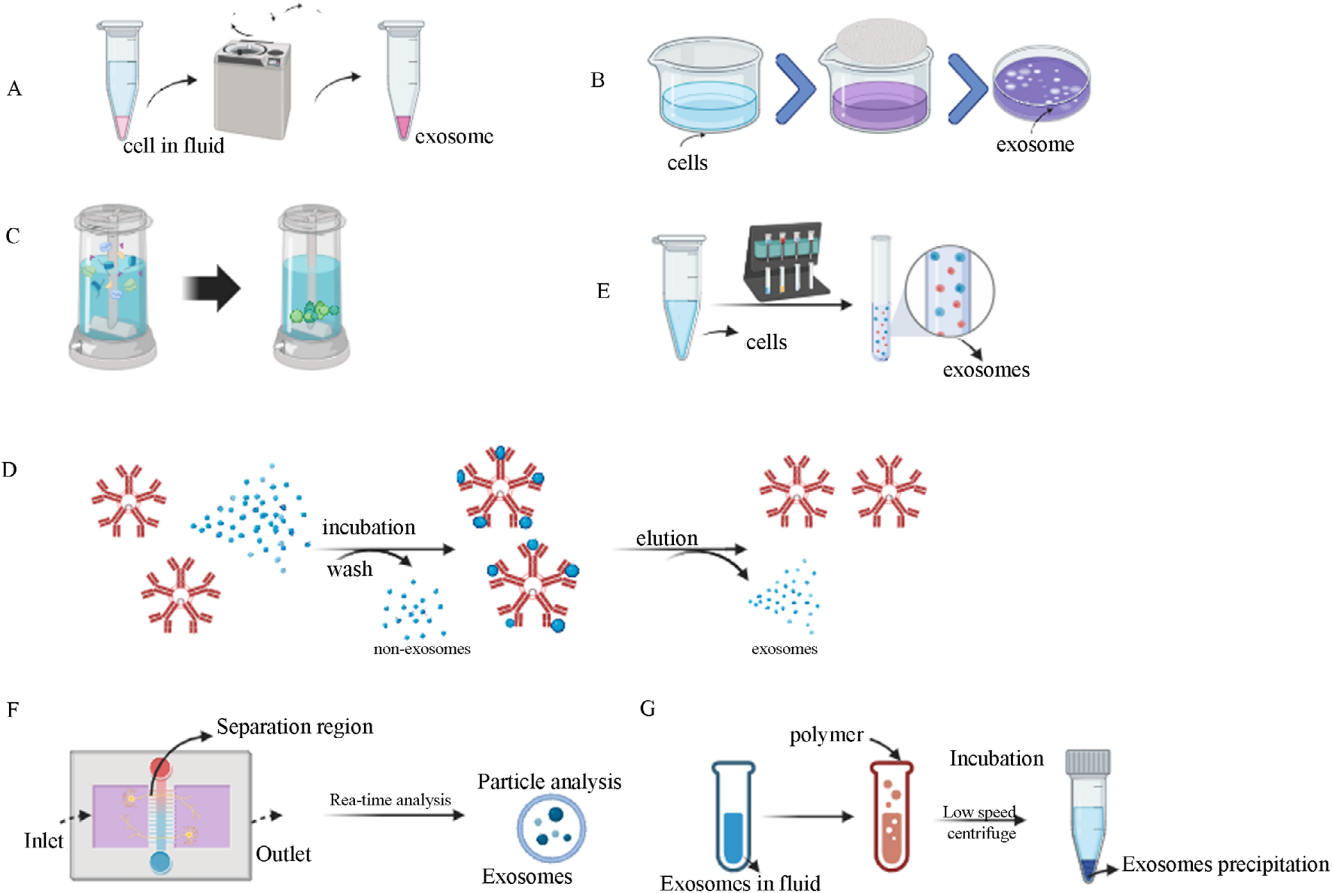
Extraction methods of exosomes. (A) Ultracentrifugation; (B) ultrafiltration; (C) size‐exclusion chromatography; (D) immunoaffinity; (E) reagent kit extraction method; (F) microfluidic technology; (G) polymer precipitation.

Ultracentrifugation, capable of producing centrifugal forces up to 1,000,000 × *g*, is a widely used method for isolating exosomes from bacteria, yeast, plants, and human cells [35, 36]. This method is widely regarded as the gold standard for exosome extraction in research due to its high yield and widespread applicability. However, it has some limitations. Ultracentrifugation can cause exosomes damage due to the high shear forces involved, leading to compromised membrane integrity and functionality [37]. Two common variants of ultracentrifugation are differential ultracentrifugation (DUC) and density gradient ultracentrifugation (DGUC) (Figure 2A).DUC involves progressively separating smaller particles from larger ones through a series of increasingly intensive steps over extended periods [38, 39]. While DUC is straightforward, it is prone to contamination with non‐exosomal proteins. A primary drawback of this method is the contamination with non‐exosomal proteins, such as liposomes, which often co‐sediment with exosomal particles due to their comparable density. DGUC separates vesicles or smaller particles by inert media with different densities and specific centrifugal forces [40]. When exosomes move at a certain speed, they will stay in a region that matches their density. This method offers high separation efficiency, purity, and preserves membrane integrity [41]. However, both methods still face challenges such as particle aggregation and contamination with proteins and nucleic acids. Even if the modified scheme is superior to DUC, there are still problems of particle aggregation and protein and nucleic acid contamination. The basic principle behind ultracentrifugation is that, when a centrifugal force acts on a non‐uniform mixture, particles with a higher density and size relative to others will sediment earlier. Ultracentrifugation is crucial for isolating viruses, organelles, bacteria, and small extracellular vesicles in exosomes centrifugation [42].

#### 2.1.6 Ultrafiltration

Ultrafiltration employs membrane separation technology to isolate exosomes from cell culture media or biological fluids [43]. Typically, membranes with pore sizes of 0.1–0.8 μm are used to separate smaller exosomes and other particles. Exosomes were separated by filtration membranes with different pore sizes (Figure 2B). Ultrafiltration can be used to process large volumes while avoiding sample disruption caused by high‐speed centrifugation. The advantages of ultrafiltration are speed, simplicity, economy, and the production of a large number of proteins and RNA, while the disadvantages are mainly low purity and exosomes structural differences in torsion and extrusion.

#### 2.1.7 Size‐Exclusion Chromatography (SEC)

Size exclusion chromatography (SEC) is frequently employed for the separation and purification of exosomes (Figure 2C). The advantages of this method are lower pollutant levels and homogeneity of exosomes. Exosomes can be isolated and purified from various biological fluids such as plasma, serum, urine, cerebrospinal fluid, saliva, milk, and tears. The method operates by eluting larger particles first, followed by smaller vesicles, and then a non‐membrane‐bound protein cationic polymer, using the starting biofluid as the mobile phase and a porous gel filter as the stationary phase [44]. SEC offers advantages such as lower contamination levels and high homogeneity of exosomes. However, it often requires combination with other methods, such as ultracentrifugation, to achieve higher purity. The stationary phase or chromatographic column (sepacrylic acid) may include various gel polymers like crosslinked dextran (Sephadex), agarose, polyacrylamide (Biogel P), or allyl dextran. Gel chromatography relies on the size‐based separation of particles using gel materials under specific conditions [45]. This method is often used in combination with ultracentrifugation and uses various materials such as polyacrylamide gels and agarose gels.

#### 2.1.8 Immunoaffinity

The mechanism of the method is to match the affinity link between the antibody and the surface protein. This method is mainly used to isolate biomarkers that adhere to the surface of exosome membranes. This method uses specific antibodies immobilized on affinity columns or magnetic beads to extract target molecules (such as exosomes) specifically [46] (Figure 2D). Immunoaffinity is frequently employed alongside DUC to enhance the quality of isolated exosomes. The main disadvantage of this method is that the use of antibodies can only collect exosomes recognized by antibodies, but it does result in high yields. Failure to remove the antibody from the vesicle post‐precipitation can compromise exosomes integrity [47]. While this method offers high specificity and sensitivity, its effectiveness is constrained by the availability of appropriate antibodies. Since its use requires a large number of antibody coupling beads, its high price may also limit its application. Therefore, it may only be applicable to studies with small sample sizes, which limits its scope of application.

#### 2.1.9 Reagent Kit Extraction Method

The basic principle is to use a polymer network to collect exosomes of a certain size, which are then recovered by low‐speed centrifugation. This method offers a high extraction yield and can extract exosomes from any biological fluid. However, the disadvantage is that it is expensive and difficult to apply in clinical and basic experiments (Figure 2E).

#### 2.1.10 Microfluidic Technology

It is a size‐based separation chip, which is used for the separation and extraction of exosomes [48]. The basic principle is to use nanoporous membranes of different sizes and pressure‐driven fluid flow to separate exosomes (Figure 2F). The extraction of exosomes by this method mainly relies on continuous filtration of nano‐ultrafiltration [49]. This method may lead to contamination by non‐exosomal humoral peptides like alpha1‐antitrypsin and albumin, and result in decreased yield and purity, particularly affecting the purity of exosomal cargo such as RNA and miRNA [50]. The primary cause of decreased exosome purity and yield is attributed to the interaction between vesicles and cell membranes, which serve as binding surfaces for exosomes and proteins, leading to aggregate formation and pore blockage [51, 52].

#### 2.1.11 Polymer Precipitation

This method operates by affecting the solubility or dispersibility of highly hydrophilic polymers through exosomes. Exosomes can be precipitated by changing their solubility. The sample was combined with a polymer‐based precipitation solution, incubated at 4°C, and subjected to low‐speed centrifugation. Polyethylene glycol (PEG) is frequently utilized for polymer precipitation (Figure 2G). The method is straightforward, suitable for large sample volumes, and offers high yield, facilitating the clinical application of exosomes [53]. However, the size range of isolated exosomes matches that of DUC, leading to decreased purity and specificity due to the co‐precipitation of soluble non‐exosomal proteins. This approach may contaminate the final exosomes with polymers and positively charged molecules, potentially hindering subsequent omics‐based studies [54, 55].

#### 2.1.12 Magnetic‐Based Isolation

Antibody coated magnetic beads are an effective method for cell and vesicle isolation based on high affinity antibody–antigen reaction with high recovery and purity [56]. The exosomes isolated by traditional magnetic beads can be rapidly characterized by flow cytometry [57]. Zhao et al. developed a microfluidic chip that combines immunomagnetic separation and continuous flow mixing. The method captures exosomes from plasma by binding magnetic beads to antibodies, and the continuous flow operation provides a scalable sample volume for on‐chip analysis. The labeled combination samples are quantified multiple times to increase the separation speed. The advantages of this method are that it can improve the multiple quantization speed of the label combination, and the preparation is simple and low cost, which can be applied to point‐of‐care detection (POCT) [58].

### 2.2 Emerging Technologies and Future Directions

While traditional methods such as ultracentrifugation and ultrafiltration remain widely used, emerging technologies are addressing their limitations. For example, advancements in microfluidics and nanotechnology are enabling high‐throughput, high‐purity exosomes isolation with minimal damage [59]. Additionally, novel approaches such as genetically engineered cells and changing the cell culture conditions are showing promise for improving yield and scalability [60]. Despite these advancements, challenges such as standardization, scalability, and cost‐effectiveness need to be addressed to facilitate the translation of these technologies into clinical practice.

In summary, exosomes extraction methods have certain limitations, and the selection of different methods should be based on specific experimental requirements and research goals (Table 1). When using various exosomes extraction methods, it is important to avoid interference from cell debris, microparticles, and other contaminants to ensure a higher purity and stability of the extracted samples.

**TABLE 1:** Exosomes extraction methods and their advantages and disadvantages.

Extraction methods	Mechanism	Advantages	Disadvantages	Reference
Ultracentrifugation	The components have different sizes and densities	Simple and efficient, it can separate samples on a large scale	The purity is moderate, the yield is low, exosomes may be damaged	[61]
Ultrafiltration	Particles of varying sizes and molecular masses	Simple operation, high component purity, moderate yield	Exosomes with tiny diameters may be lost due to blockages on the filtration membrane	[62]
Size‐exclusion chromatography (SEC)	Different sizes of particles and molecular densities	It has high yield and purity	There is a potential for protein contamination, requiring the use of special columns and packaging	[63]
Immunoaffinity	Interaction of antibodies with specific exosome membrane proteins	High specificity, sensitivity and high purity	Expensive, limited by the availability of suitable antibodies	[64]
Reagent Kit Extraction Method	Exosomes of a certain size are collected using a polymer network and then recovered by low‐speed centrifugation.	The extraction rate is high and exosomes can be extracted from any biological sample solution.	Expensive, not suitable for experimental and clinical use.	
Microfluidic Technology	The fluid passes through a microchannel, trapping exosomes based on surface markers	The extraction rate is high and the applicability is wide	Only suitable for small samples, and there is a possibility of loss of exosomes during washing	[65]
Polymer precipitation	Exosomes have an effect on the solubility or dispersion of highly hydrophilic polymers.	High yield, ideal for large volume samples.	Due to the influence of co‐purified protein polymers or residual polymers, the purity level is low.	[64]
Magnetic‐baced isolation	Antibody–antigen high‐affinity reaction	High purity, simple preparation, low cost, can be used in point‐of‐care detection (POCT)	The efficiency and recovery rate are low, and the exogenic activity is easily affected by PH and salinity	[56]

## 3 Loading Methods of Exosomes for Drug Delivery

Modifying exosomes and loading drugs into them is essential for their application as drug delivery vehicles. Especially in recent years, with the deepening understanding of exosomes, their special targeting ability and difficulty in degradation in vivo have made exosomes a hot research topic for treating related diseases in various fields. Although the method of loading drugs into exosomes is still in the exploratory stage, some methods are relatively mature, including direct incubation, ultrasound, electroporation, and repeated freeze–thaw cycles.

### 3.1 Direct Incubation Method

**FIGURE 3:**
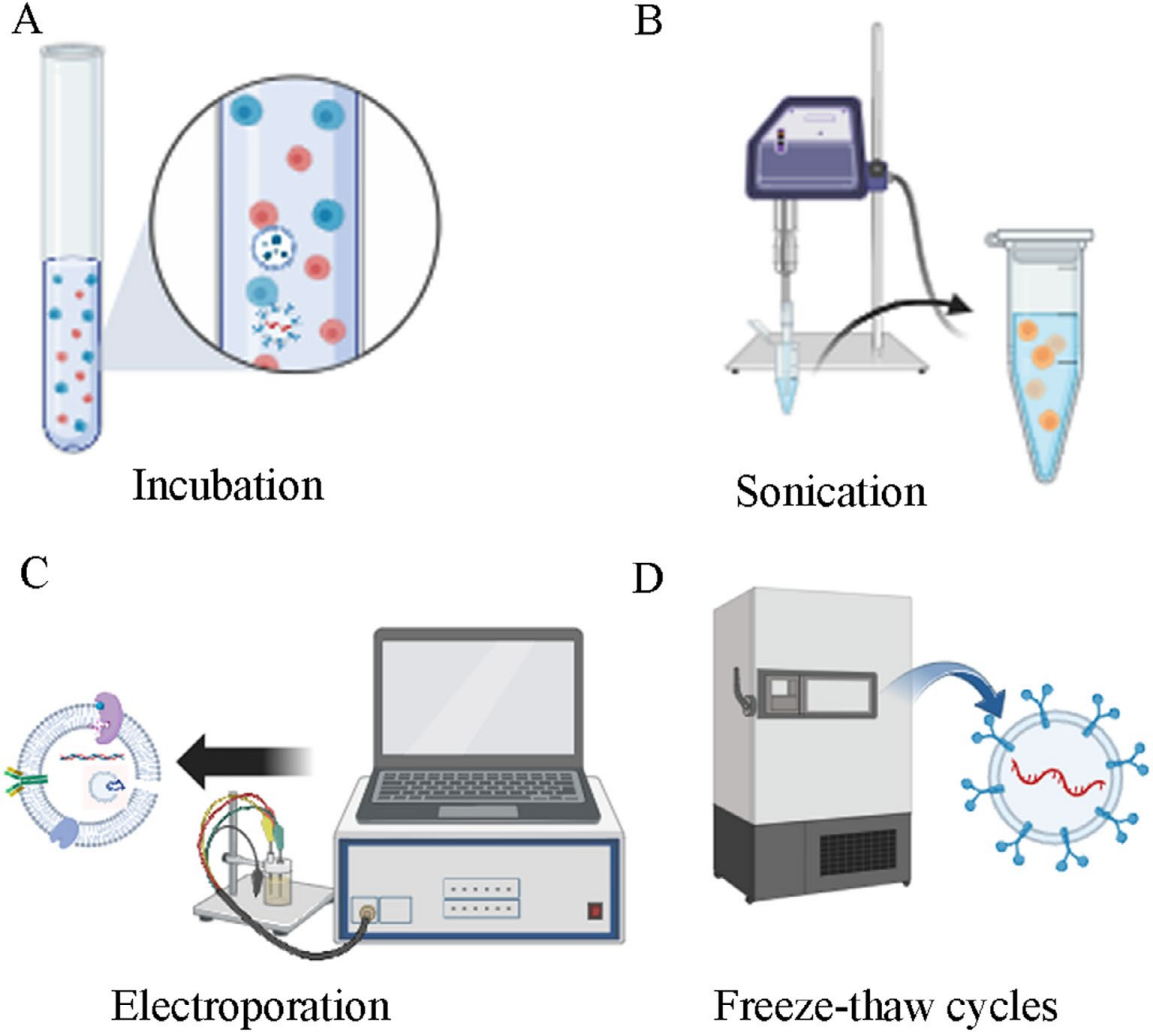
Loading methods of exosomes for drug delivery. (A) Direct incubation method; (B) ultrasound method; (C) electroporation method; (D) repeated freeze–thaw method.

This involves co‐incubating the target drug with the parent cells at room temperature, and loading some of the target drugs in the cell cytoplasm into exosomes through endocytosis [66]. Another way is to directly co‐incubate the target drug with exosomes and then extract the exosomes containing the target drug (Figure 3A). This method preserves the integrity and function of exosomes well, requires relatively low experimental equipment, and has strong repeatability, but the loading efficiency is relatively low.

### 3.2 Ultrasound Method

The basic principle is to apply ultrasound energy using a probe to reduce the toughness of the exosomes membrane, increasing the transport capacity of drugs (Figure 3B). However, this method requires high experimental equipment and damages the membrane structure, increasing the risk of target drug extravasation and leading to undesirable drug loading amounts.

### 3.3 Electroporation Method

The basic principle is to apply an electric current to create pores on the exosomes membrane, increasing the transport capacity of drugs [67, 68] (Figure 3C). However, this method is prone to damage membrane integrity, and high‐pressure pulses can also destroy protein structures, causing increased exosomes aggregation and reduced exosomes activity.

### 3.4 Repeated Freeze–Thaw Method

This involves freezing the target drug and exosomes in a −80°C freezer or liquid nitrogen, repeating this process for three cycles [66, 69] (Figure 3D). The basic principle is that after repeated cycles of freezing and thawing, the exosomes membrane becomes more permeable, allowing more drugs to enter the exosomes and increase drug intake. This method is easy to perform and highly repeatable, but has limited loading efficiency, may change the size of exosomes, and may lead to exosomes aggregation (Table 2).

**TABLE 2:** Loading methods for exosomal delivery of drugs.

Loading method	Mechanism	Advantage	Disadvantage	Reference
Direct incubation	The drug is introduced into exosomes according to the concentration gradient during incubation	It is limited to one specific type of drug, and the amount released after incubation is not sufficient for clinical trials	The sample loading efficiency is low	[70]
Ultrasound	The ultrasonic energy applied by the probe reduces the toughness of the exosome membrane and increases the drug transport capacity	Loading efficiency is higher than electroporation and incubation	High requirements on experimental equipment, and damage to membrane structure, increase the risk of target drug extravasation, resulting in unsatisfactory drug loading	
Electroporation	An electric current is applied to form holes in the exosome membrane, thereby increasing the drug's transport capacity	The method can increase the loading of hydrophilic small molecules in exosomes and increase the efficiency of RNA in exosomes	It is easy to destroy the integrity of the membrane, and high pressure pulse will also destroy the protein structure, resulting in increased exosome aggregation and decreased exosome activity	[71]
Freeze–thaw cycle	After repeated freeze–thaw cycles, the permeability of exosome membranes increases, allowing more drugs to enter exosomes and increasing drug intake	The method is simple to operate and has high repeatability	Loading efficiency is limited, may change the size of exosomes, and may lead to exosome aggregation	[72]

The structural characteristics of exosomes make them have obvious advantages as drug carriers. Several studies have focused on drug loading into exosomes [73]. Current research has established that drug loading efficiency varies with different loading techniques. For example, when Paclitaxel(PTX) is loaded with Raw264.7 cell‐derived exosomes, the efficiency of Direct incubation, Electroporation, and Sonication is 1.4 (SEM ± 0.38%), 5.3 (SEM ± 0.48%), and 28.29 (SEM ± 1.38%), respectively [74]. The efficiency of different drugs varies even when using the same method. The loading efficiency of exosomes derived from Raw 264.7 Macrophages (mouse) is 8.0%–11.0% when Doxorubicin is loaded by Sonication [75]. In addition, the exosome loading efficiency of different cell sources is not completely consistent. Exosomes derived from LNCaP and PC‐3 cells were loaded with paclitaxel, achieving a direct incubation efficiency of 9.2% (SD ± 4.5%) [76]. The efficiency of paclitaxel loading in exosomes from Milk (bovine) origin was 7.9% ± 1.0% [77]. The efficiency of loading Doxorubicin in Immature dendritic cells (mouse) derived exosomes by Electroporation was < 20% [78] (Table 3).

**TABLE 3:** Loading content and Efficiency.

EVs sources	Content	Method	Efficiency	Reference
Raw 264.7	PTX	Direct incubation Electroporation Sonication	1.4(SEM ± 0.38%) 5.3 (SEM ± 0.48%) 28.29 (SEM ± 1.38%)	[74]
LNCaP and PC‐3	PTX	Direct incubation	9.2% (SD ± 4.5%)	[76]
Milk	PTX	Direct incubation	7.9% ± 1.0%	[77]
Immature dendritic cells	Dox	Electroporation	< 20%	[78]
Raw 264.7	Dox	Sonication	8.0%–11.0%	[75]

## 4 Tumor Treatment and the Occurrence of Tumor Drug Resistance

Currently, the main methods of treating malignant tumors are drug therapy, radiation therapy, laser therapy, and molecular biology‐based selective therapy, with drug therapy remaining the primary method. However, most patients experience disease progression and treatment failure after receiving drug therapy, and one of the main reasons for this phenomenon is drug resistance. Currently, there is no clear reason for tumor drug resistance, but some possible mechanisms include the following:

### 4.1 Tumor Heterogeneity

The different genotypes of cells within the same tumor lead to phenotypic differences, which are known as tumor heterogeneity [79]. Anything that affects tumor heterogeneity can lead to drug resistance.

### 4.2 Tumor Microenvironment

The tumor microenvironment of a solid tumor consists primarily of diverse cells, blood vessels, and the extracellular matrix [80]. Numerous studies have shown that the tumor microenvironment is related to the drug resistance of malignant tumors.

### 4.3 Tumor Stem Cells

Tumor stem cells initiate tumor formation and may be the main cause of tumor drug resistance. The metastasis and recurrence of tumors are the results of tumor stem cells escaping from drug killing [81]. The development of multidrug resistance in tumors may be due to the ability conferred by tumor stem cells.

### 4.4 Inactivation of Anti‐Tumor Drugs

Some anti‐tumor drugs require metabolic activation to function, such as ara‐C, a commonly used drug for treating acute myeloid leukemia, which needs to be catalyzed by deoxycytidine kinase into cytarabine triphosphate to exert its effect. Studies have shown that mutations or downregulation of deoxycytidine kinase can lead to drug resistance [82, 83, 84, 85].

### 4.5 Reduced Drug Uptake and Increased Efflux

Methotrexate, a commonly used chemotherapy drug, is a drug that relies on reduced folate carrier (RFC) to enter cells [86]. RFC is an 85 kDa membrane protein, and when its expression is reduced or inhibited, drug uptake decreases [87]. Anthracycline antibiotics, plant alkaloids, and anthrapyrazoles are common chemotherapy drugs for which P‐gP is a substrate. The expression of P‐gP in tumor cells mainly responds to the induction of drugs. When the cytotoxicity of chemotherapy drugs affects tumor cells, the expression of P‐gP increases, playing a protective role and resulting in chemotherapy drug resistance [88].

### 4.6 Changes in Drug Metabolism

When anti‐tumor drugs act on cells, the functions of cell detoxification and repair systems are enhanced, leading to rapid inactivation of drugs and allowing damaged DNA in tumor cells to be repaired [89].

### 4.7 Mutations of Drug Target Genes

In the process of anti‐tumor treatment, as time passes, tumor cells may undergo mutations in target genes to adapt to targeted drugs, leading to treatment resistance [90, 91].

### 4.8 Changes in the Tumor Immune System

Chemotherapy drugs can reduce tumor cell apoptosis by inducing immune tolerance, causing tumor cells to evade immune surveillance, and leading to resistance to tumor treatment [92].

## 5 Application of Exosomes in Cancer Treatment

In response to the resistance mechanisms of tumors, appropriate anti‐tumor drugs have been developed clinically. However, due to the heterogenicity of the drugs themselves and changes in the tumor microenvironment, clinical efficacy is not always optimistic. Therefore, developing low immunogenicity drugs that do not produce an immune response is important for improving tumor resistance. Exosomes, originating from natural immune cells, possess distinct membrane proteins unlike other carriers [93]. CD9 and CD81 facilitate exosome fusion with recipient cells, CD55 and CD59 prevent non‐specific complement pathway attacks, and CD47 shields exosomes from macrophage recognition and phagocytosis [94]. Consequently, these exosomes exhibit remarkable features, including biocompatibility, biodegradability, stability, and low immunogenicity, making them effective in anti‐tumor therapy [95, 96]. Due to their lack of immunogenicity, they do not trigger immune responses, allowing them to evade immune system clearance and remain in the body and tissues for extended periods. Studies indicate that exosomes can transport drugs across the blood–brain barrier, targeting intracranial lesions and enhancing the treatment of primary and secondary brain diseases [97]. In addition, exosomes from different tissue cell sources have been shown to have tissue selectivity, and the different proteins carried on the surface of exosomes often determine their targeting ability in vivo. The exosome formation and release processes are influenced by cellular stress states and the tumor microenvironment, causing variations in surface protein composition and affecting their targeting ability. Research has shown that oncolytic viruses are a recognized cancer treatment, but they must be used locally because of the rapid immune clearance rate [98]. If the virus is encapsulated in cancer exosomes, it can be delivered and targeted to the tumor microenvironment. Macrophage exosomes can also be used to deliver the anti‐tumor drug to tumors [99, 100]. Li et al. identified a T7 peptide‐decorated exosome‐based nanocarrier system for the delivery of Galectin‐9 siRNA to stimulate macrophage repolarization in glioblastoma [101]. Exosomes derived from macrophages can also regulate the functions of immune cells. For example, exosomes derived from M1‐type macrophages can activate immune cells such as T cells and natural killer (NK) cells, thereby enhancing the immune response [102]. DNA recombinants may even target exosomes to enhance immunogenicity. The expression vector encoding the antigen fusion CD63 can transfer antigens to exosomes in vivo, triggering a stronger anti‐tumor response in the same‐gene lymphoma model [103]. Liu J et al. demonstrated that macrophages targeting Glypican‐3 and delivering drug‐loaded exosomes provide effective cytotherapy in mouse models of solid tumors [104]. Due to their tumor‐oming properties and other characteristics, exosomes derived from mesenchymal stem cells (MSCs) have shown potential in the treatment of pancreatic cancer. Recent studies have shown that mesenchymal stem cells (MSCs) can be induced to migrate by tumor‐derived growth factors, form spheroids with pancreatic cancer cells in vitro, and also increase the angiogenesis of orthotopic pancreatic tumors. At the same time, MSCs can target the Wnt signaling pathway to affect the tumor cell cycle. MSCs engineered to carry anti‐cancer drugs can enhance the tumor suppression effect, reduce side effects, improve the efficacy of chemotherapy, and improve the quality of life of patients [105]. In lung cancer, which has a relatively high mortality rate, exosomes have shown great potential in aspects such as the diagnosis of lung cancer, the transformation of lung cancer treatment, and the delivery of drugs for lung cancer [106]. Exosomes can also serve as biomarkers for cancer diagnosis and prognosis. Studies have found that specific microRNAs (miRNAs) in exosomes, such as miR‐1268b and miR‐6075, possess high sensitivity and specificity in the early detection of various cancers, including lung cancer and pancreatic cancer. They can be used as promising diagnostic and prognostic biomarkers, which is helpful for the early detection of cancer and the monitoring of the disease condition [107].

Currently, numerous clinical trials are being conducted on exosomes for tumor treatment. The study titled A Measure of the Expression of the HER2‐HER3 Dimer in Tumor and Blood (Exosomes) Samples From Patients With HER2 Positive Breast Cancer Receiving Clinical Trial of HER2 Targeted Therapies (NCT04288141) is investigating HER2‐positive breast cancer. Participants undergo blood tests and receive anti‐HER2 therapy. Investigators will also take cancer samples from biopsies with permission. The study employed the FLM‐FRET technique to assess signal pairing in both tumor and blood samples. The researchers will measure whether the signal pairing levels in the two samples are the same, which could make it possible for a blood test to be used in patients who choose to receive anti‐HER2 treatment, thereby reducing patient suffering. Alterations in signal pairing can potentially forecast treatment resistance. In pancreatic cancer, iExosomes in Treating Participants With Metastatic Pancreas Cancer With KrasG12D Mutation Clinical trials are underway. This study evaluated the optimal dosage and side effects of mesenchymal stromal cell‐derived KrasG12D siRNA exosomes for treating patients with metastatic KrasG12D mutated pancreatic cancer. Additionally, Aethlon Medical Inc. conducted an early feasibility study (EFS) named Hemopurifier Plus Pembrolizumab in Head and Neck Cancer, which investigated the use of Hemopurifier with pembrolizumab (Keytruda) to eliminate immunosuppressive exosomes in a first‐line treatment setting. In Lung cancer, Richeng Jiang and colleagues are conducting clinical trials called Serum Exosomal miRNA Predicting the Therapeutic Efficiency in Lung Squamous Carcinoma. This study aimed to evaluate serum exosome miRNAs as biomarkers for predicting the efficacy of combined immunotherapy and chemotherapy. In rectal cancer, Andrew Hoover, University of Kansas Medical Center, has conducted a clinical trial called Exosomes in Rectal Cancer. This study aimed to investigate exosomal biomarker levels in patients with locally advanced rectal cancer undergoing neoadjuvant chemoradiotherapy. The study compared the pathological response rates of APR and LAR with exosome expression rates before and after chemoradiotherapy. Exosomes are extensively utilized in oncology for both tumor diagnosis and treatment with exosome‐loaded drugs. Clinical trials further demonstrate their broad applicability and potential across various tumor types. Numerous institutions and hospitals are conducting or have completed clinical trials on exosome applications in tumors (Table 4). As these studies conclude, the resulting data will significantly enhance the understanding of exosomes in tumor diagnosis and treatment. Exosomes are anticipated to serve as clinical drug delivery tools for direct application in tumor treatment.

**TABLE 4:** Ongoing clinical trials with exosomes.

NCT number	Study title	Study status	Stage	Conditions	Sponsor	Study type
NCT05854030	Serum Exosomal miRNA Predicting the Therapeutic Efficiency in Lung Squamous Carcinoma	Recruiting	—	Lung neoplasm|Squamous cell carcinoma|Exosomes	Tianjin Medical University Cancer Institute and Hospital	Observational
NCT03102268	ncRNAs in Exosomes of Cholangiocarcinoma	Unknown	—	Cholangiocarcinoma|Benign biliary stricture	The Second Hospital of Nanjing Medical University	Observational
NCT04288141	A Study to Measure the Expression of the HER2‐HER3 Dimer in Tumor and Blood (Exosomes) Samples From Patients With HER2 Positive Breast Cancer Receiving HER2 Targeted Therapies	Recruiting	—	HER2‐positive breast cancer	King's College London	Observational
NCT04530890	Interest of Circulating Tumor DNA in Digestive and Gynecologic/Breast Cancer	Recruiting	Phase: not application	Breast cancer digestive cancer gynecologic cancer circulating tumor DNA exosomes	Poitiers University Hospital	Interventional
NCT05625529	ExoLuminate Study for Early Detection of Pancreatic Cancer	Recruiting	—	Pancreas cancer exosomes extracellular vesicles pancreatic neoplasms	Biological Dynamics	Observational
NCT03874559	Exosomes in Rectal Cancer	Unknown	—	Rectal cancer	University of Kansas Medical Center	Observational
NCT06026735	Non‐small Cell Lung Cancer With Central Nervous System Metastasis	Recruiting	—	Lung cancer with central nervous system metastasis	National Taiwan University Hospital	Observational
NCT03109873	Metformin Hydrochloride in Affecting Cytokines and Exosomes in Patients With Head and Neck Cancer	Completed	Phase: early phase 1	Larynx lip oral cavity pharynx	Sidney Kimmel Cancer Center at Thomas Jefferson University	Interventional
NCT04453046	Hemopurifier Plus Pembrolizumab in Head and Neck Cancer	Terminated	Phase: not application	Squamous cell carcinoma of the head and neck	Aethlon Medical Inc.	Interventional
NCT01159288	Trial of a Vaccination With Tumor Antigen‐loaded Dendritic Cell‐derived Exosomes	Completed	Phase: phase 2	Non small cell lung cancer	Gustave Roussy, Cancer Campus, Grand Paris	Interventional
NCT03542253	Combined Diagnosis of CT and Exosome in Early Lung Cancer	Unknown	—	Early lung cancer	Second Affiliated Hospital of Soochow University	Observational
NCT01779583	Circulating Exosomes As Potential Prognostic And Predictive Biomarkers In Advanced Gastric Cancer Patients (“EXO‐PPP Study”)	Unknown	—	Gastric cancer	Hospital Miguel Servet	Observational
NCT03608631	iExosomes in Treating Participants With Metastatic Pancreas Cancer With KrasG12D Mutation	Active_Nor_Recruiting	Phase: phase 1	KRAS NP_004976.2:p.G12D metastatic pancreatic adenocarcinoma pancreatic ductal adenocarcinoma Stage IV Pancreatic Cancer AJCC v8	M.D. Anderson Cancer Center	Interventional
NCT01668849	Edible Plant Exosome Ability to Prevent Oral Mucositis Associated With Chemoradiation Treatment of Head and Neck Cancer	Completed	Phase: phase 1	Head and neck cancer oral mucositis	University of Louisville	Interventional
NCT05286684	Feasibility of Exosome Analysis in Cerebrospinal Fluid During the Diagnostic Workup of Metastatic Meningitis (Exo‐LCR)	Recruiting	Phase: not application	Breast cancer	Centre oscar lambret	Interventional
NCT01294072	Study Investigating the Ability of Plant Exosomes to Deliver Curcumin to Normal and Colon Cancer Tissue	Recruiting	Phase: not application	Colon cancer	University of Louisville	Interventional
NCT02310451	Study of Molecular Mechanisms Implicated in the Pathogenesis of Melanoma. Role of Exosomes	Completed	Phase: not application	Metastatic melanoma	Centre Hospitalier Universitaire de Nice	Interventional
NCT04427475	Prediction of Immunotherapeutic Effect of Advanced Non‐small Cell Lung Cancer	Unknown	Phase: not application	NSCLC patients	Fudan University	Interventional
NCT04227886	Study on Predictive Biomarkers of Neoadjuvant Chemoradiotherapy for Rectal Cancer	Unknown	—	Rectal neoplasm malignant carcinoma chemoradiotherapy neoadjuvant therapy predictive biomarkers adenocarcinoma	Fudan University	Observational
NCT03974204	Analyses of Exosomes in the Cerebrospinal Fluid for Breast Cancer Patients With Suspicion of Leptomeningeal Metastasis.	Withdrawn	Phase: not application	Breast cancer leptomeningeal metastasis	Centre Oscar Lambret	Interventional
NCT04499794	The Study of Exosome EML4‐ALK Fusion in NSCLC Clinical Diagnosis and Dynamic Monitoring	Recruiting	—	Untreated advanced NSCLC patients FISH identified ALK fusion positive or negative	Chinese Academy of Medical Sciences	Observational
NCT06381648	Detecting Lymph Node Metastasis in Intrahepatic Cholangiocarcinoma (LyMIC)	Recruiting	—	Cholangiocarcinoma Cholangiocarcinoma, Intrahepatic Cholangiocarcinoma Resectable Cholangiocarcinoma; Liver	City of Hope Medical Center	Observational
NCT04298398	Impact of Group Psychological Interventions on Extracellular Vesicles in People Who Had Cancer	Unknown	Phase: not application	Cancer	Instituto Portugues de Oncologia, Francisco Gentil, Porto	Interventional
NCT05575622	Clinical Study for Combined Analysis of CTC and Exosomes on Predicting the Efficacy of Immunotherapy in Patients With Hepatocellular Carcinoma	Recruiting	—	Hepatocellular carcinoma	Zhongnan Hospital	Observational
NCT04852653	A Prospective Feasibility Study Evaluating Extracellular Vesicles Obtained by Liquid Biopsy for Neoadjuvant Treatment Response Assessment in Rectal Cancer	Recruiting	—	Rectal cancer liquid biopsy	University Hospital, Bordeaux	Observational
NCT04653740	Omic Technologies to Track Resistance to Palbociclib in Metastatic Breast Cancer	Unknown	Phase: not application	Advanced Breast Cancer	Centre Oscar Lambret	Interventional
NCT02862470	Anaplastic Thyroid Cancer and Follicular Thyroid Cancer‐derived Exosomal Analysis Via Treatment of Lovastatin and Vildagliptin and Pilot Prognostic Study Via Urine Exosomal Biological Markers in Thyroid Cancer Patients	Completed	—	Thyroid Cancer	National Taiwan University Hospital	Observational

### 5.1 Safety Evaluation of Exosome‐Based Therapies

The safety of exosome‐based therapies is a critical consideration for clinical translation. While exosomes are generally considered biocompatible and low in immunogenicity, their potential to induce immune responses or off‐target effects cannot be overlooked [108]. For example, exosomes derived from certain cell types, such as cancer cells, may carry oncogenic cargo that could promote tumor progression or metastasis [109]. Additionally, the presence of residual contaminants from isolation procedures, such as proteins or lipoproteins, may trigger adverse immune reactions [110].

To address these concerns, rigorous safety evaluations are essential. Preclinical studies should include comprehensive toxicity assessments, such as in vitro cytotoxicity assays and in vivo biodistribution studies, to evaluate the potential risks of exosome‐based therapies [111]. Furthermore, the development of standardized protocols for exosome characterization, including purity, stability, and cargo composition, is crucial to ensure safety and reproducibility [112]. Recent advances in analytical techniques, such as mass spectrometry and single‐vesicle imaging, have improved our ability to assess exosome safety and quality [113].

### 5.2 Design Methods of Exosomes for Drug Delivery

Exosomes are an ideal platform for drug cultivation due to their properties [95, 114, 115]. Despite the advantages of natural exosomes, their clinical application is limited by low targeting capability and insufficient concentration of functional molecules [116]. Engineered exosomes refer to the modification and optimization of exosomes through biotechnological means to enhance their natural properties or endow them with new functions [115]. In recent years, significant progress has been made in the field of engineered exosomes, particularly in the expression of specific surface markers. For example, through genetic engineering of parent cells, exosomes can be designed to express targeting ligands (such as RGD peptides or transferrin receptors) or immune‐modulating molecules (such as PD‐L1), enabling precise targeted delivery or immunotherapy [117]. Additionally, chemical modification techniques have been widely applied to exosome surfaces, such as using click chemistry to conjugate antibodies or aptamers to enhance their targeting capabilities and functionality [118]. These advancements have demonstrated the immense potential of engineered exosomes in cancer therapy, neurological diseases, tissue repair, and vaccine development. To address these limitations, exosome design can be achieved through two primary methods: parental cell‐based exosome engineering and direct exosome engineering. The key distinction between these approaches lies in whether the genetic engineering process is conducted prior to or following exosome isolation. The former is categorized into two types. In the first category, that is, in a non‐specific manner, this is done by transfecting parent cells with a plasmid or analog of interest. The second class consists of two subgroups focused on the specific loading of molecules: exosome surface display and lumen loading. Exosome surface display utilized various fusion proteins and signal peptides, including Lamp2b (lysosomal‐associated membrane protein 2b) [119], tetrapesterins (CD63, CD9, CD81) [120], GPI (glycophosphoproteinlipoinositol) [121], PDGFRs (platelet‐derived growth factor receptors) [122], lactate adhesion (C1C2 domain) [123], VSVG (vesicular stomatitis virus glycoprotein) [124] and other signal peptides. By fusing a protein of interest on a signaling peptide, the protein will be presented on the surface of the exosome. The basis for embedding molecules in exosomal cavities is based on molecular classification modules (MSMs). Various methods for using different MSMs exist, including engineered ubiquitin tags [125], WW tags [126], non‐functional mutants of HIV‐I Nef protein [127], and RNA‐binding modules for loading RNA into exosomes [128], among others. In direct exosome engineering, exosomes can be designed by means of electroporation, ultrasonic treatment, incubation, biological coupling, freeze–thaw and extraction [129]. It is believed that with the advancement of technology, an increasing number of advanced methods will emerge to provide better prospects for drug delivery using exosomes. Recent advancements in this field have further expanded the toolbox for exosome engineering. For instance, CRISPR/Cas9 technology has been employed to genetically modify parent cells, enabling the production of exosomes with enhanced targeting capabilities and therapeutic payloads [130]. Moreover, advancements in microfluidic‐based methods have enabled high‐throughput and precise loading of therapeutic cargo into exosomes, addressing the challenge of insufficient functional molecule concentration [131]. Despite these promising developments, several challenges remain. For example, the scalability of engineered exosome production, the potential for off‐target effects, and the long‐term stability of modified exosomes in vivo need to be thoroughly investigated [132]. Furthermore, regulatory and safety considerations must be addressed to facilitate the translation of these innovative approaches into clinical practice. It is believed that with the continuous advancement of technology, an increasing number of advanced methods will emerge, providing better prospects for drug delivery using exosomes.

### 5.3 Comparative Analysis of Exosomes and Other Nanocarrier Systems

Exosomes offer several advantages over synthetic nanocarriers, such as liposomes and polymeric nanoparticles, for drug delivery. Unlike synthetic systems, exosomes are naturally derived and exhibit high biocompatibility, low immunogenicity, and the ability to cross biological barriers, including the blood–brain barrier [64, 133]. Additionally, exosomes can be engineered to display specific surface markers, enhancing their targeting capabilities. However, exosomes also face challenges that synthetic nanocarriers do not. For instance, the scalability and reproducibility of exosome production are more complex compared to the well‐established manufacturing processes for liposomes and polymeric nanoparticles [134]. Furthermore, synthetic nanocarriers can be precisely tailored in terms of size, surface charge, and drug release kinetics, whereas exosomes exhibit inherent heterogeneity that complicates standardization [135]. Despite these challenges, exosomes hold unique potential for personalized medicine due to their ability to carry endogenous cargo and interact with recipient cells in a biologically relevant manner [136]. Future research should focus on combining the strengths of exosomes and synthetic nanocarriers to develop hybrid systems that maximize therapeutic efficacy while minimizing limitations.

### 5.4 Application of Exosomes in Small Molecule Anti‐Tumor Drug Transport

Currently, research on exosome transport of small molecule anti‐tumor drugs mainly focuses on anthracyclines and taxanes. Tian et al. employed genetic engineering to derive immature dendritic cells (IMDCs) from mice expressing the Lamp2b iRGD peptide, which is specific to av. integrins. Exosomes were then isolated from these IMDCs and used to transport docetaxel [78]. The research demonstrated that iRGD‐modified exosomes selectively targeted av. integrin‐expressing breast cancer cells, leading to inhibited tumor growth [137]. Another common method of transporting small molecule anti‐tumor drugs is taxane drugs. Studies indicate that mesenchymal stem cell‐derived exosomes can encapsulate and transport paclitaxel, exhibiting potent anti‐proliferative effects on human pancreatic cancer cells when loaded with taxane drugs [138]. Kim et al. reported that macrophage‐derived exosomes loaded with paclitaxel exhibit enhanced cytotoxicity in p‐gp‐positive resistant MDCK (canine kidney) cells compared to the free drug [139].

### 5.5 Application of Exosomes in Large Molecule Drugs

Due to the limited molecular weight and composition of large molecule drugs, they are easily digested and degraded in the tumor microenvironment. To reach the target lesion site, they need to pass through a series of barriers and obstacles. Exosomes serve as intercellular carriers capable of crossing barriers effortlessly, highlighting their significant role in transporting large molecule drugs. Haney et al. reported that catalase‐loaded exosomes were administered intranasally, resulting in significant accumulation in the brains of Parkinson's mice. This demonstrates that exosomes can effectively deliver catalase to target neurons, offering neuroprotective effects against oxidative stress [140].

### 5.6 Application of Exosomes in Anti‐Tumor Immunotherapy

The immunotherapeutic potential of exosomes was first demonstrated in 1998 [141]. The exploration of exosomes as functional communication pathways, such as in antigen presentation and costimulatory molecules, has led to the development of a novel class of exosome‐based cancer immunotherapy. The discovery of additional immunological and oncogenic characteristics of exosomes will lead to the identification of new therapeutic targets and mechanisms. Exosomes participate in tumor immune cell cross‐talk, providing many opportunities for tumor immunotherapy. While exosome‐based immunotherapy remains unapproved, numerous clinical trials have commenced in recent years.

Exosomes from immunocytes, particularly those derived from dendritic cells, are promising for cancer immunotherapy due to their capacity to elicit tumor‐specific immune responses [142]. DC‐exosomes carrying interferon‐gamma enhance this effect by increasing NK (nature kill) cell secretion of interferon‐gamma and tumor necrosis factor. Incorporating melanoma antigen 1 (MART1), a T cell‐recognized antigen, into interferon‐gamma‐enhanced exosomes could potentially activate tumor‐specific responses in NK cells; however, its impact on humans is minimal [143]. Enhancing alpha‐fetoprotein (AFP) expression in DC‐exosomes boosts tumor immunity. AFP enhances the aggregation of MHC I, MHC II, and costimulatory molecules on DC‐exosomes, transforming the tumor microenvironment from immune suppression to stimulation by boosting T cell infiltration and decreasing Treg cell presence [144]. The incorporation of ovalbumin, lipopolysaccharide, and interferon‐gamma into DC‐exosomes facilitates the conversion of immunosuppressive M2 macrophages into immune‐activating M1 macrophages, enhances antigen presentation by DCs, and directly stimulates T cells [145].

Exosomes derived from NK cells and macrophages are potential candidates for immunotherapy [146]. NK cell exosomes contain FasL and tumor necrosis factor, giving them cytotoxic effects against cancer cells [147]. Exosomes produced from the cell membrane of NK cells also exhibit these characteristics. Enhancing tumor attack can be achieved by activating NK cells using IL‐15 [148]. This therapy enhances the expression of TRAIL and the activation of NKp46 and NKp30 receptors on NK cell exosomes, leading to improved tumor targeting and cytotoxicity [149].

Another common cancer treatment method is exosomes derived from human HEK293 cell lines [150]. HEK293 exosomes are engineered with signal regulatory protein alpha (Sirpα) to inhibit CD47 at the tumor site, enhancing macrophage‐mediated phagocytosis of tumor cells and promoting CD8^+^ T cell infiltration into the tumor [151]. HEK293 exosomes are capable of incorporating PH20 hyaluronidase to degrade high molecular weight hyaluronic acid (HA) within the tumor microenvironment. Exosomes derived from PD‐1‐expressing HEK293 cell membranes target tumor PD‐L1 and transport indoleamine 2,3‐dioxygenase‐1 inhibitors to the tumor microenvironment, thereby decreasing Treg levels and enhancing anti‐tumor responses [152]. To reduce the uptake of HEK293 exosomes by the liver and thus increase circulation time and localization to the tumor site, glucosyl sulfate is used to block the A‐type scavenger receptor (SR‐A) on exosomes, enhancing their performance [153]. Synthetic exosomes are engineered to mimic the tumor‐targeting and immune‐stimulating functions of natural exosomes by modifying cell membrane usage. Leukocyte membranes are collected and squeezed into vesicles for coating microspheres. Melanoma cell membranes are similarly used to produce PEGylated nanovesicles that induce anti‐tumor immune responses [154]. SMART‐ExOs are engineered with tumor antigen and CD3 to trigger specific immune responses [155].

### 5.7 Challenges in Clinical Translation of Exosome‐Based Therapies

Despite the significant progress in exosome research and engineering, translating these findings into clinical practice remains a formidable challenge. Key barriers include issues related to scalability, reproducibility, and regulatory compliance, which must be addressed to realize the full potential of exosome‐based therapies.

#### 5.7.1 Scalability

One of the primary challenges in clinical translation is the scalable production of exosomes. Current isolation methods, such as ultracentrifugation and ultrafiltration, are labor‐intensive and yield limited quantities of exosomes, making them unsuitable for large‐scale clinical applications [156]. Emerging technologies, such as microfluidic‐based isolation and bioreactor systems, offer promising solutions by enabling high‐throughput exosome production with consistent quality [157]. For example, bioreactors can support the continuous culture of exosome‐producing cells, significantly increasing yield while maintaining exosome integrity [158]. However, optimizing these technologies for industrial‐scale production remains a work in progress.

#### 5.7.2 Reproducibility

Reproducibility is another critical issue, as exosome heterogeneity and variability in isolation methods can lead to inconsistent therapeutic outcomes. Standardization of exosome isolation, characterization, and storage protocols is essential to ensure batch‐to‐batch consistency [112]. Initiatives such as the Minimal Information for Studies of Extracellular Vesicles (MISEV) guidelines provide a framework for reporting exosome research, but widespread adoption and implementation are still needed [159]. Additionally, advanced analytical tools, such as single‐exosome profiling and multi‐omics approaches, can help characterize exosome subpopulations and ensure reproducibility [160, 161].

#### 5.7.3 Regulatory Hurdles

Regulatory challenges also pose significant barriers to the clinical translation of exosome‐based therapies. Exosomes are classified as biological products, and their complex nature complicates the regulatory approval process. Key concerns include the characterization of exosome composition, safety, and efficacy, as well as the development of standardized quality control measures [112]. Regulatory agencies, such as the FDA and EMA, are actively working to establish guidelines for exosome‐based products, but clear and universally accepted standards are still lacking. Collaborative efforts between researchers, clinicians, and regulatory bodies are essential to address these challenges and facilitate the translation of exosome therapies into clinical practice.

### 5.8 Potential Solutions and Future Directions

To overcome these barriers, a multi‐faceted approach is required. First, investment in scalable production technologies, such as bioreactors and microfluidic systems, is critical to meet the demand for clinical‐grade exosomes [162]. Second, the development of standardized protocols and reference materials can improve reproducibility and facilitate regulatory approval. Finally, interdisciplinary collaboration and data sharing are essential to advance the field and address unresolved challenges.

In conclusion, while exosome‐based therapies hold immense promise, significant challenges remain in their clinical translation. Addressing issues related to scalability, reproducibility, and regulatory compliance will require concerted efforts from researchers, clinicians, and industry stakeholders. By overcoming these barriers, exosome‐based therapies can be transformed from experimental tools into mainstream clinical treatments.

## 6 Conclusions and Future Perspectives

Drug resistance is a huge hurdle in daily clinical work. Exosomes have recently been identified as key modulators of resistance to anti‐cancer therapies through various mechanisms. Exosomes are nanoscale vesicles crucial for intercellular communication, influencing the function and fate of recipient cells. Numerous studies have shown that changes in the quantity and composition of exosomes in various diseases can serve as potential biomarkers for diagnosis and prognosis. Exosomes can be engineered as carriers to deliver therapeutic molecules to specific cells and tissues, offering a novel approach for disease treatment. However, due to the lack of standardized separation techniques that go beyond subcellular size, origin, and floating density, the current evaluation of exosome heterogeneity remains a major challenge. Investigating exosome heterogeneity is crucial for enhancing our comprehension of their significant role as drug carriers. Exosome heterogeneity arises from differences in their biogenesis, cellular origin, and cargo composition, leading to diverse subpopulations with distinct biological functions [163]. This variability significantly impacts their performance as drug delivery vehicles. For instance, exosomes derived from different cell types (e.g., mesenchymal stem cells vs. cancer cells) exhibit varying surface protein profiles, which influence their targeting specificity and biodistribution [9]. Exosomes from cancer cells often carry tumor‐specific markers, such as EGFR or HER2, which can enhance their ability to target tumor cells but may also limit their applicability to other diseases [15].

Recent studies have highlighted the role of exosome surface proteins in mediating cell‐specific interactions, emphasizing the need for precise engineering to optimize targeting efficiency [164].

Moreover, the heterogeneity in exosome size and membrane composition affects drug loading efficiency. Smaller exosomes tend to have higher drug encapsulation efficiency due to their larger surface‐to‐volume ratio, while larger exosomes may carry more complex cargo but face challenges in cellular uptake [165, 166]. Additionally, the presence of specific surface proteins, such as tetraspanins (CD9, CD63, CD81), can influence the binding and internalization of exosomes by recipient cells, further complicating their therapeutic application [167]. Recent advances in exosome engineering have demonstrated that modifying these surface proteins can enhance drug loading and delivery efficiency [168].

The clinical implications of exosome heterogeneity are profound. Variability in exosome subpopulations can lead to inconsistent therapeutic outcomes, as different exosome populations may exhibit varying stability, immunogenicity, and pharmacokinetic properties [169]. For example, exosomes rich in immunomodulatory molecules may suppress immune responses, while those carrying pro‐inflammatory cargo could exacerbate inflammation [170]. These factors must be carefully considered when designing exosome‐based drug delivery systems to ensure reproducibility and efficacy. Recent studies have also shown that exosome heterogeneity plays a critical role in cancer progression and therapy resistance, highlighting the need for personalized exosome‐based treatments [171].

Emerging technologies, such as single‐exosome analysis and high‐resolution imaging, are providing new insights into exosome heterogeneity and its functional consequences [172]. These tools enable researchers to characterize exosome subpopulations at the single‐particle level, paving the way for the development of more precise and personalized exosome‐based therapies. However, challenges remain in standardizing these techniques and translating them into clinical practice.

Exosome‐based therapies represent a promising frontier in drug delivery, with the potential to revolutionize the treatment of various diseases, including cancer, neurodegenerative disorders, and cardiovascular conditions. However, significant challenges remain in translating these therapies from the laboratory to the clinic.

To advance the field, future research should focus on the following areas:

1. Scalable Production Technologies: Develop cost‐effective and high‐throughput methods for exosome isolation and engineering, such as bioreactor systems and microfluidic platforms.
2. Standardization and Characterization: Establish standardized protocols for exosome isolation, characterization, and quality control to ensure reproducibility and safety.
3. Targeted Engineering: Explore novel strategies for engineering exosomes with enhanced targeting capabilities, such as CRISPR/Cas9‐mediated modification and surface ligand conjugation.
4. Hybrid Nanocarriers: Investigate the development of hybrid systems that combine the strengths of exosomes and synthetic nanocarriers to overcome existing limitations.
5. Clinical Trials: Conduct well‐designed clinical trials to evaluate the safety, efficacy, and pharmacokinetics of exosome‐based therapies in diverse patient populations.

By addressing these challenges and pursuing these research directions, exosome‐based therapies can be transformed from experimental tools into mainstream clinical treatments.

In conclusion, exosome heterogeneity is a critical factor that influences their function, targeting, and therapeutic efficacy. A deeper understanding of this variability, coupled with advances in separation and characterization technologies, will be essential for harnessing the full potential of exosomes as drug delivery vehicles.

### 6.1 Current Hot Topics in Oncology

Exosomes play a critical role in cancer biology and therapy, particularly in the context of the tumor microenvironment (TME), immunotherapy, and drug resistance [173]. Recent studies have highlighted the involvement of exosomes in mediating communication between tumor cells and stromal cells, thereby promoting tumor progression and metastasis [174]. For example, exosomes derived from cancer‐associated fibroblasts (CAFs) can transfer pro‐tumorigenic factors, such as miRNAs and proteins, to cancer cells, enhancing their invasive potential [175]. In the field of cancer immunotherapy, exosomes have emerged as promising tools for enhancing anti‐tumor immune responses. Tumor‐derived exosomes can carry tumor antigens and immune modulatory molecules, making them potential candidates for cancer vaccines [176]. Additionally, engineered exosomes loaded with immune checkpoint inhibitors or cytokines have shown promise in preclinical studies for overcoming immune evasion [177]. Exosomes also play a significant role in drug resistance, a major challenge in cancer therapy. Tumor cells can release exosomes containing drug efflux pumps or anti‐apoptotic proteins, which confer resistance to chemotherapy and targeted therapies [178]. Understanding the mechanisms underlying exosome‐mediated drug resistance may lead to the development of novel strategies to overcome this barrier. Recent advancements in exosome research, such as single‐vesicle analysis and multi‐omics approaches, are providing new insights into these complex processes [179]. These technologies enable researchers to dissect the role of exosomes in cancer biology at an unprecedented level of detail, paving the way for the development of more effective therapeutic strategies.

The recently hot topics also include: (1) The current cancer treatment methods function by inducing apoptosis or directly causing cell damage. Understanding the resistance mechanism of cancer cells to apoptosis can explain why the treatment of certain malignant tumors fails. At present, based on the understanding of the apoptosis process, new therapies are being developed to induce the apoptosis of cancer cells while limiting the killing of normal cells [180]. (2) During the clonal expansion of normal epithelial cells, the TNF‐a signal is provided by immune cells in the surrounding microenvironment and other factors. It promotes the proliferation of cells carrying cancer‐elated gene mutations and plays a key role in the early stage of cancer. After the formation of cancer, some cancer cells will autonomously produce TNF‐a. This self‐roduced TNF‐a will further promote the invasion of cancer cells into the surrounding tissues, which is a key step in the development and spread of tumors. Therefore, the TNF‐a signaling pathway provides new targets and possibilities for the early detection and treatment of cancer in the advanced stage [181]. (3) The tumor microenvironment (TME) is crucial for cancer progression, metastasis, and treatment response. The epithelial‐esenchymal transition (EMT) process is induced by TME signals, which can enhance the motility and invasiveness of cancer cells, promoting cancer progression and metastasis. Disrupting the promoting effect of TME on EMT by targeting various components of TME is a promising cancer treatment strategy, which helps improve the treatment efficacy, address the problem of treatment resistance, and provide a more nuanced approach to cancer treatment [182]. (4) Cytotoxic T lymphocytes (CTLs) play a crucial role in anti‐tumor immunity. It includes not only the traditional CD8+ CTLs, but also various subsets such as CD4+ T cells, NK cells, and γδ T cells [183].

Exosomes offer superior safety, biocompatibility, barrier‐crossing capability, targeting, and reduced immunogenicity compared to traditional drug carriers, indicating their promising potential. However, there are still many obstacles to using exosomes as a clinically common drug carrier. First, the quality of exosomes cannot be guaranteed due to the complex extraction steps and lack of efficient extraction processes. Each step is affected by various factors, and slight differences in a step can result in significant changes in the quality and purity of exosomes obtained. Second, the storage of exosomes is also a challenge as they are unstable. Storing at −80°C greatly increases the difficulty and cost of transportation, while storage at −4°C affects their structure and quality. Third, effective drug loading methods differ from those of liposomes. Exosomes are complete vesicles before drug loading, and various ways and means of transporting drugs into exosomes destroy the integrity and function of exosome structures to some extent. Therefore, optimizing drug loading methods is necessary.

Although research on exosome drug‐loading is still in its early stages, achievements in related fields have shown promise. Advancements in technology and methods will likely lead to the widespread clinical application of exosome drug‐loading in tumor treatment, enhancing cancer patient survival and prognosis. Future research should also investigate the diversity and heterogeneity of exosomes across various biological environments to explore potential interactions and synergies with other cellular exosomes. Exosome research should integrate with emerging fields like nanotechnology, bioengineering, and artificial intelligence to address health issues with personalized solutions.

## Author Contributions

**Panpan Feng:** writing original draft, investigation, editing/revising. **Jian Gao** and **Xiaodong Zhang:** review, supervision. **Yan Li** and **Lei Jiang:** editing, review, and funding. All authors read and approved the submitted version.

## Ethics Statement

The authors have nothing to report.

## Consent

The authors have nothing to report.

## Conflicts of Interest

The authors declare no conflicts of interest.

## Data Availability

Data sharing was not applicable to this review as no data sets were generated and analyzed in this study.
